# Supply-chain strategies for essential medicines in rural western Kenya during COVID-19

**DOI:** 10.2471/BLT.20.271593

**Published:** 2021-02-10

**Authors:** Dan N Tran, Phelix M Were, Kibet Kangogo, James A Amisi, Imran Manji, Sonak D Pastakia, Rajesh Vedanthan

**Affiliations:** a Department of Pharmacy Practice, Temple University School of Pharmacy, Philadelphia, United States of America (USA).; b Academic Model Providing Access to Healthcare, Eldoret, Kenya.; c Department of Family Medicine, Moi University School of Medicine, Eldoret, Kenya.; d Department of Pharmacy, Moi Teaching and Referral Hospital, Eldoret, Kenya.; e Department of Pharmacy Practice, Purdue University College of Pharmacy, 640 Eskenazi Ave, West Lafayette, IN 46202, USA.; f Department of Population Health, New York University Grossman School of Medicine, New York, USA.

## Abstract

**Problem:**

The coronavirus disease 2019 (COVID-19) pandemic has disrupted health systems worldwide and threatened the supply of essential medicines. Especially affected are vulnerable patients in low- and middle-income countries who can only afford access to public health systems.

**Approach:**

Soon after physical distancing and curfew orders began on 15 March 2020 in Kenya, we rapidly implemented three supply-chain strategies to ensure a continuous supply of essential medicines while minimizing patients’ COVID-19 exposure risks. We redistributed central stocks of medicines to peripheral health facilities to ensure local availability for several months. We equipped smaller, remote health facilities with medicine tackle boxes. We also made deliveries of medicines to patients with difficulty reaching facilities.

**Local setting:**

Τo implement these strategies we leveraged our 30-year partnership with local health authorities in rural western Kenya and the existing revolving fund pharmacy scheme serving 85 peripheral health centres.

**Relevant changes:**

In April 2020, stocks of essential chronic and non-chronic disease medicines redistributed to peripheral health facilities increased to 835 140 units, as compared with 316 330 units in April 2019. We provided medicine tackle boxes to an additional 46 health facilities. Our team successfully delivered medications to 264 out of 311 patients (84.9%) with noncommunicable diseases whom we were able to reach.

**Lessons learnt:**

Our revolving fund pharmacy model has ensured that patients’ access to essential medicines has not been interrupted during the pandemic. Success was built on a community approach to extend pharmaceutical services, adapting our current supply-chain infrastructure and working quickly in partnership with local health authorities.

## Résumé

**Problème:**

La pandémie de maladie à coronavirus 2019 (COVID-19) a bouleversé les systèmes de santé du monde entier et menacé l'approvisionnement en médicaments essentiels. Dans les pays à faible et moyen revenu, les patients vulnérables ayant uniquement accès aux soins de santé publics ont été particulièrement affectés.

**Approche:**

Peu après l'instauration de la distanciation physique et du couvre-feu le 15 mars 2020 au Kenya, nous avons rapidement mis en œuvre trois stratégies visant à assurer un approvisionnement continu en médicaments essentiels, tout en limitant les risques d'exposition des patients au coronavirus. Nous avons redistribué les principaux stocks de médicaments aux établissements sanitaires périphériques afin de garantir leur disponibilité pendant plusieurs mois. Nous avons fourni des boîtes de matériel médical aux petits centres de soins implantés dans des régions reculées. Nous avons également livré des médicaments aux patients incapables de se rendre dans un établissement.

**Environnement local:**

Pour déployer ces stratégies, nous avons profité de nos trente années de partenariat avec les autorités sanitaires locales dans les régions rurales du Kenya occidental et compté sur le modèle existant de financement pharmaceutique renouvelable, qui dessert 85 centres de soins périphériques.

**Changements significatifs:**

Les stocks de médicaments essentiels servant au traitement de maladies chroniques et non chroniques redistribués aux centres de soins périphériques sont passés de 316 330 unités en avril 2019 à 835 140 unités en avril 2020. Nous avons procuré des boîtes de matériel médical à 46 centres de soins supplémentaires. Notre équipe a réussi à livrer des médicaments à 264 des 311 patients (84,9%) souffrant de maladies non transmissibles que nous sommes parvenus à contacter.

**Leçons tirées:**

Grâce à notre modèle de financement pharmaceutique renouvelable, les patients ont pu accéder aux médicaments essentiels sans interruption durant la pandémie. Ce succès repose sur une approche communautaire destinée à étendre les services pharmaceutiques en adaptant l'infrastructure de notre chaîne d'approvisionnement actuelle, et en avançant rapidement par le biais de partenariats avec les autorités sanitaires locales.

## Resumen

**Situación:**

La pandemia de la enfermedad por coronavirus 2019 (COVID-19)ha perturbado los sistemas sanitarios de todo el mundo y ha amenazado el suministro de medicamentos esenciales. Se ven especialmente afectados los pacientes vulnerables de los países de ingresos bajos y medios que solo pueden acceder a los sistemas sanitarios públicos.

**Enfoque:**

Poco después de que comenzaran el distanciamiento físico y las órdenes de toque de queda el 15 de marzo de 2020 en Kenia, pusimos en marcha rápidamente tres estrategias para garantizar un suministro continuo de medicamentos esenciales y minimizar al mismo tiempo los riesgos de exposición de los pacientes al COVID-19. Redistribuimos las existencias centrales de medicamentos a los centros de salud periféricos para garantizar la disponibilidad local durante varios meses. Equipamos a los centros de salud más pequeños y remotos con cajas de botiquín. También hicimos entregas de medicamentos a pacientes con dificultades para llegar a los centros.

**Marco regional:**

Para poner en práctica estas estrategias, hemos aprovechado nuestra asociación de 30 años con las autoridades sanitarias locales de las zonas rurales del oeste de Kenia y nos hemos apoyado en el modelo existente de financiación farmacéutica rotatoria, que atiende a 85 centros de salud periféricos.

**Cambios importantes:**

Las existencias de medicamentos esenciales para el tratamiento de enfermedades crónicas y no crónicas redistribuidas a los centros de atención periférica pasaron de 316.330 unidades en abril de 2019 a 835.140 unidades en abril de 2020. Hemos adquirido cajas de material médico para otros 46 centros de salud. Nuestro equipo pudo entregar medicamentos a 264 de los 311 pacientes (84,9%) con enfermedades no transmisibles con los que logramos contactar.

**Lecciones aprendidas:**

Gracias a nuestro modelo de financiación farmacéutica rotatoria, los pacientes pudieron acceder a los medicamentos esenciales sin interrupción durante la pandemia. Este éxito se basa en un enfoque comunitario para ampliar los servicios farmacéuticos adaptando nuestra infraestructura de cadena de suministro existente y avanzando rápidamente mediante asociaciones con las autoridades sanitarias locales.

## ملخص

المشكلة لقد أدت جائحة مرض فيروس كورونا إلى تعطيل النظم الصحية على مستوى العالم وهددت الإمداد بالأدوية الأساسية. من المتأثرين بشكل خاص المرضى المهمشون في البلدان ذات الدخل المنخفض والمتوسط ممن يستطيعون أن يتحملوا تكلفة التعامل مع النظم الصحية العامة فقط.

الأسلوب بمجرد أن بدأت أوامر التباعد الجسدي وحظر التجول في يوم 15 مارس/آذار 2020 في كينيا، اتبعنا سريعًا ثلاث استراتيجيات لضمان الإمداد المستمر بالأدوية الأساسية مع الحد من مخاطر تعرض المرضى لفيروس كورونا. لقد أعدنا توزيع المخزونات الأساسية من الأدوية على المنشآت الصحية الطرفية لضمان التوافر المحلي لعدة أشهر. قمنا بتجهيز منشآت صحية نائية أصغر بحاويات الأدوية. قمنا أيضًا بعمليات توصيل للأدوية إلى المرضى الذين يعانون من صعوبة الوصول إلى المنشآت.

المواقع المحلية لتنفيذ هذه الاستراتيجيات، اعتمدنا على شراكتنا الممتدة لفترة 30 سنة مع السلطات الصحية المحلية في غرب كينيا الريفي والمخطط الدوار القائم لتمويل الصيدليات والذي يخدم 85 مركزًا صحيًا طرفيًا.

التغيّرات ذات الصلة في شهر أبريل/نيسان 2020، ارتفعت مخزونات الأدوية الأساسية للأمراض المزمنة وغير المزمنة التي تمت إعادة توزيعها على المنشآت الصحية الطرفية إلى 835140 وحدة في مقابل 316330 وحدة في شهر أبريل/نيسان 2019. لقد وفّرنا حاويات أدوية إلى 46 منشأة صحية إضافية. نجح فريقنا في توصيل العلاجات إلى 264 من 311 مريضًا (84,9%) مصابين بأمراض غير معدية استطعنا الوصول إليهم.

الدروس المستفادة لقد ضمن نموذجنا الدوار لتمويل الصيدليات عدم انقطاع حصول المرضى على الأدوية الأساسية أثناء الجائحة. اعتمد النجاح على نهج مجتمعي لتمديد الخدمات الصيدلانية وتكييف بنيتنا التحتية الحالية لسلسلة الإمداد والعمل سريعًا في شراكة مع السلطات الصحية المحلية.

## 摘要

**问题:**

新型冠状病毒肺炎疫情扰乱了全球卫生系统,并威胁到基本药物的供应。中低收入国家那些仅可负担公共卫生系统就诊费用的弱势患者群体所受影响尤其严重。

**方法:**

自 2020 年 3 月 15 日肯尼亚开始实施物理距离限制措施及宵禁令之后,我们随即推出了三种供应链战略,以确保不间断供应基本药物,同时最大程度地降低患者感染冠状病毒肺炎的风险。我们将中央库存药物重新分配给次级卫生机构,以保证在随后几个月内地方药物供应充足。我们为规模较小、位置偏远的卫生机构配备了医疗用品箱。我们还为难以前往卫生机构的患者配送药物。

**当地状况:**

为实施这些战略,我们充分利用我们与肯尼亚西部农村地区地方卫生当局 30 年来建立的合作关系并切实推行为 85 个次级卫生中心提供服务的现有循环基金药房计划。

**相关变化:**

与 2019 年 4 月(316,330 份)相比,2020 年 4 月重新分配给次级卫生机构的基本慢性和非慢性疾病库存药物已增至 835,140 份。我们为另外 46 个卫生机构提供了医疗用品箱。在 311 名我们能接触到的患有非传染性疾病的患者中,我们团队成功为 264 名患者(占 84.9%)提供了药物治疗。

**经验教训:**

我们的循环基金药房模型确保了大流行期间患者仍能够不间断地获得基本药物。本次能够取得成功,主要在于扩大了社区的药学服务范围,调整了我们当前的供应链基础设施并与当地卫生部门迅速展开了合作。

## Резюме

**Проблема:**

Пандемия заболевания, вызываемого коронавирусом 2019-nCoV, негативно отразилась на системах здравоохранения во всем мире и создала угрозу для поставок основных лекарственных средств. Особенно значительное влияние было оказано на уязвимых пациентов в странах с низким и средним уровнем доходов, которые могут позволить себе только услуги системы общественного здравоохранения.

**Подход:**

Вскоре после того, как 15 марта 2020 года в Кении были введены физическое дистанцирование и комендантский час, нами были незамедлительно реализованы три стратегии, касающиеся цепочек поставок, в целях обеспечения непрерывных поставок основных лекарственных средств при минимизации риска заражения пациентов коронавирусом. Нами были перераспределены центральные запасы лекарственных средств по периферийным медицинским учреждениям для обеспечения их наличия на местах в течение нескольких месяцев. Небольшие отдаленные медицинские учреждения были оборудованы аптечками. Кроме того, нами осуществлялась доставка лекарственных средств пациентам, не имеющим возможности беспрепятственно добираться до лечебных учреждений.

**Местные условия:**

Для реализации указанных стратегий нами были использованы механизмы, наработанные за 30 лет нашего партнерства с местными органами здравоохранения в сельских районах Западной Кении, а также существующий оборотный фонд аптечной индустрии, обслуживающий 85 медицинских центров, находящихся на периферии.

**Осуществленные перемены:**

В апреле 2020 года запасы основных лекарственных средств от хронических и нехронических заболеваний, перераспределенные в периферийные медицинские учреждения, увеличились до 835 140 единиц в сравнении с показателем в 316 330 единиц, зафиксированным в апреле 2019 года. Дополнительные 46 медицинских учреждений были обеспечены аптечками. Наша команда успешно поставила лекарства 264 пациентам из 311 (84,9%) с неинфекционными заболеваниями, до которых нам удалось добраться.

**Выводы:**

Наша модель аптечного оборотного фонда обеспечила беспрерывный доступ пациентов к основным лекарственным средствам во время пандемии. Успех был основан на социологическом подходе к расширению доступности фармацевтических услуг, адаптации нашей текущей инфраструктуры цепочки поставок и оперативном взаимодействии с местными органами здравоохранения.

## Introduction

The coronavirus disease 2019 (COVID-19) pandemic has challenged health systems worldwide as they cope with the demands of infection control and management of the disease while maintaining the delivery of other ongoing essential care services to patients.^1^^,^^2^ Low- and middle-income countries such as Kenya face new threats to an already overburdened health system.^1^ One important challenge of the pandemic is the threat to the availability of essential medicines. Containment and mitigation strategies, central to the COVID-19 response, have the potential to disrupt medication supplies for vulnerable patients who can only afford access to the public-sector health system.^1^^,^^2^ This supply-chain disruption is caused by government-mandated lockdowns, limitation of essential clinical services, lack of personal protective equipment for health-care providers, and stock-outs of essential medicines.^1^^,^^3^^,^^4^ Addressing these challenges is essential to ensuring a robust response for patients with COVID-19-related needs, while preserving care for patients with other acute and chronic illnesses. In this paper, we describe strategies used to respond to these challenges, to implement proactive and patient-centric solutions, and to secure a continuous supply of essential medicines to patients in rural western Kenya.

## Local setting

The Academic Model Providing Access to Healthcare partnership in western Kenya has developed care, education and research infrastructure to respond to the needs of patients living with human immunodeficiency virus infection.^5^ The partnership has subsequently leveraged this infrastructure for the management of a comprehensive set of noncommunicable diseases.^6^^,^^7^ A key component of the chronic disease management programme has been the creation, implementation and scale-up of the revolving fund pharmacy scheme, in partnership with the appropriate local health authorities and local community leadership. Over the past decade the revolving fund pharmacy model has successfully addressed many supply-chain needs for essential medicines in alignment with the Kenya essential medicines list.^8^^–^^10^ The scheme operates within the catchment area of the Academic Model Providing Access to Healthcare partnership, spanning across seven counties and serving a population of 8 million people in western Kenya.

After the first case of COVID-19 was identified in Kenya on 13 March 2020, the government immediately implemented physical distancing and sheltering-in-place orders beginning 15 March 2020. Before this time, the revolving fund pharmacy scheme used a pull-based supply system whereby medicines were supplied to 85 peripheral health facilities throughout western Kenya based on drug order requests from those health facilities. However, after the Kenyan government enacted physical distancing guidelines and curfew requirements, we switched to a push-based supply system to support more rapid availability of medicines during this health crisis. We developed three context-specific strategies to ensure a continuous, timely and secure supply of essential medicines to patients throughout western Kenya. We present the available data on 33 essential medicines in 14 therapeutic categories.

## Approach

The first strategy was the creation of decentralized warehouses in peripheral health facilities. In response to the physical distancing directives, staff of the revolving fund pharmacy scheme worked in partnership with appropriate county health authorities to determine the types and quantities of medications, as well as a delivery plan for essential medicines. Before the COVID-19 pandemic, the majority of the revolving fund pharmacy scheme’s medications were stored in a central pharmacy warehouse. After March 2020, we decentralized the medication supply by redistributing the central stock of essential medications for chronic disease management (such as hypertension, diabetes and epilepsy) and acute ailments (such as infectious diseases) to 11 health facilities throughout western Kenya.

The second strategy was to provide patients with safer access to essential medicines. As of 15 March 2020, patients with chronic disease needs were encouraged to limit visits to hospitals. In response, staff of the chronic disease management programme increased the time between follow-up clinical visits. The change in follow-up interval was communicated by the clinicians to patients and the staff of the revolving fund pharmacy, who then modified the quantity of medications provided to each patient. Thus, we could easily refill patients’ long-term medications without unnecessarily exposing patients or clinicians to the risk of COVID-19. We converted many of the health facilities in the chronic disease management network into sites where medicine tackle boxes containing a full complement of chronic disease medications were provided to assist staff with dispensing. These facilities were the smallest ones, located in very rural areas, where chronic disease medications have traditionally not been available. Medicine tackle boxes were stationed at these facilities even on days when there was no chronic disease management clinic, so that patients could come and obtain medication refills at their convenience. The clinicians in our network informed patients about the new system so that patients could correctly refill their medications at facilities equipped with medicine tackle boxes. Patients were reminded to follow all safety measures as per the health ministry guidelines, including wearing of masks and hand-hygiene. By enabling access to chronic disease medicines at the nearest health facility, we avoided the need for patients to travel long distances via public transport to reach higher-level facilities.

The third strategy was community delivery of medications. For patients who faced extra problems in reaching even their nearby health facilities (such as informal sector labourers, full-time caregivers or patients with disabilities), we developed a system of direct deliveries of medication to patients. Using our point-of-care electronic medical record system,^11^^,^^12^ we identified patients who were due for medication refills. A member of the pharmacy staff called each patient to verify demographic, clinical and medication information, and invited the patient to come to a conveniently located medication drop-off point in the community. Drop-off points could be a patient’s home, a local church, the community chief’s office, a local dispensary or any other agreed upon location that a patient could conveniently visit while adhering to social distancing recommendations. To maintain confidentiality, medications were pre-packed in opaque brown bags to conceal the contents. A 90-day supply of medication was dispensed to reduce the frequency of contact the patients were required to have with health-care staff. On distribution days, patients were given time-slots for attendance so that there were no more than six patients at any time. Patients observed hand-hygiene (washing hands when they reached the drop-off point) and physical distancing (maintaining at least 2 m of space from others) while receiving their medicines from the pharmacist who used personal protective equipment. The pharmacy staff verified the medication and gave each patient appropriate counselling to ensure therapeutic safety. We encouraged patients to pay via the local cashless mobile phone-based payment service. Patients were also advised to purchase national health insurance to take advantage of the benefits package including outpatient medication coverage. For patients who could not afford a 90-day supply of medications, we loaned them the cost of the medications using our revolving drug fund and designed a repayment plan over the course of 90 days. Patients with acute complications were evaluated by a clinician via synchronous telephone consultation, thus streamlining the referral process and avoiding unnecessary visits to the health facility.

## Relevant changes

The supply of these medications to facilities surged in April 2020, 1 month after Kenya’s first COVID-19 case was identified (Table 1). The quantity of medicines for chronic diseases supplied increased about 2.5-fold in April 2020 relative to April 2019 (from 308 760 to 787 200 units) and the quantity supplied for non-chronic diseases increased 6.3-fold (from 7570 to 47 940 units). These increases ensured that medications were available in the selected peripheral health facilities for several months. We were able to be proactive and responsive by adapting our responses to the local context during this health crisis.

**Table 1:** Essential medications supplied to health-care facilities over 4-month periods in 2019 and 2020, western Kenya

Month	Chronic disease medicines					Non-chronic disease medicines			
	No. of units supplied^a^			Relative difference^b^		No. of units supplied^a^			Relative difference^b^
	2019	2020				2019	2020		
January	583 386	642 796		1.1		33 930	58 210		1.7
February	352 136	426 682		1.2		14 620	55 585		3.8
March	489 968	705 924		1.4		13 720	26 470		1.9
April	308 760	787 200^c^		2.5		7 570	47 940^c^		6.3

^a^ 1 unit = 1 tablet, capsule, bottle or vial.

^b^ Relative difference = no. of units supplied in 2020 ÷  no. of units supplied in 2019. Data were available on 33 essential medicines in 14 therapeutic categories: 10 chronic disease medicine categories and 4 non-chronic disease medicine categories. The data are from the project’s own database.

^c^ 1 month after central medicine stocks were redistributed to 11 peripheral health facilities. The first case of coronavirus disease 2019 in Kenya was reported on 13 March 2020 and government-issued physical distancing directives in Kenya were issued on 15 March 2020.

Before March 2020, we had 11 peripheral health facilities equipped with tackle boxes. In less than 1 month after the first case of COVID-19 in Kenya, we had converted and equipped an additional 46 peripheral and rural health facilities with tackle boxes, so that more patients could easily receive their medications while minimizing COVID-19 exposure risks.

As of 19 June 2020, our team has delivered medications to 264 out of 311 patients (84.9%) whom we were able to reach. All patients received a 90-day supply of medicines at each encounter, followed by a follow-up telephone call to ensure the medications were being used correctly. Currently, our team is working closely with health authorities, local COVID-19 rapid-response teams and community strategy focal personnel. The plan is for continuous drug delivery efforts, community-based portable care delivery and community-health volunteer engagement to ensure patients do not miss any treatments for hypertension, diabetes and other noncommunicable diseases.

## Lessons learnt

Preserving the supply of essential medicines in low-resource settings is essential to protect patients with and without COVID-19-related complications during this pandemic. Our paper describes a proactive approach to ensuring the continuous, timely and secure supply of essential medicines for public-sector patient populations throughout western Kenya (Box 1). First, we have learnt that the proactive push-based supply strategy worked effectively during this time of crisis when there are immediate disruptions to the supply chain. Second, we remained flexible and forward thinking, knowing that one strategy would not work across all settings. As a result, we created multiple strategies to decentralize our medication supply. For example, medicine tackle boxes ensured close-to-home medication pick-ups for our patients. Lastly, medicine delivery to various community drop-off points required multiple points of coordination. By working closely with local health authorities, including the public health offices, we maximized patient trust, while minimizing the chance of inadvertently causing community spread of COVID-19.

Box 1 **Summary of main lessons learnt**

- Community-centric and proactive strategies ensured the continuous, timely and secure supply and availability of essential medicines for public-sector patient populations during the coronavirus disease 2019 pandemic.
- Adaptability, flexibility and forward thinking allowed us to leverage and convert the current pharmacy network to more local medicine distribution points so that patients continued to have safe access to essential medicines.
- Local partnership was required to ensure an important pillar of the health system was not interrupted during this health crisis.
